# Sepiolite and Zirconia Nanoparticles Doped with Erythrosine: Adhesive Bond Strength and Antimicrobial Performance in Primary Carious Dentin

**DOI:** 10.12669/pjms.42.8.16412

**Published:** 2026-08

**Authors:** Abeer Ali Alshami, Hiba F Kattan

**Affiliations:** 1 Abeer Ali Alshami, Department of Preventive Dental Sciences, College of Dentistry, Princess Nourah bint Abdulrahman University, P.O. Box 84428, Riyadh 11671, Saudi Arabia; 2 Hiba F Kattan, Department of Preventive Dental Sciences, College of Dentistry, Princess Nourah bint Abdulrahman University, P.O. Box 84428, Riyadh 11671, Saudi Arabia

**Keywords:** Etch and rinse adhesive, Erythrosine, Micro tensile bond strength, Sepiolite Nanoparticles, Zirconium oxide nanoparticles

## Abstract

**Objective:**

Antimicrobial effectiveness against Lactobacillus and micro tensile bond strength (μTBS) of etch and rinse adhesive (ERA) modified using Erythrosine (Ery), Ery-doped Zirconium oxide nanoparticles (ZrO_2_NPs), and Ery-doped Sepiolite Nanoparticles (SepNPs) bonded to caries-affected dentin (CAD) in primary dentition.

**Methodology:**

The present in vitro study was approved by the Institutional Review Board and Ethical Committee of Princess Nourah Binte Abdul Rahman University, IRB# 25-0063. The study duration was two months, November 20, 2025 - January 20, 2026. Primary CAD was removed. All the specimens were divided into four groups based on the application of ERA modification. Group-1 (Conventional ERA), Group-2 (Ery-modified ERA), Group-3 (Ery-doped ZrO_2_NPs modified ERA), and Group-4 (Ery-doped SepNPs modified ERA). Composite build-up was performed on all the specimens after adhesive application, and the specimens were exposed to μTBS testing and fracture analysis. All adhesives (modified and unmodified) were further tested for antimicrobial efficiency against Lactobacillus. The statistical analysis was conducted using a one-way analysis of variance (ANOVA) and a Tukey post hoc test.

**Results:**

Group-4 (Ery-doped SepNPs-ERA) treated samples displayed the minimum survival of microorganisms (0.30 ± 0.04 CFU/mL) and the highest bond integrity (15.02±0.61MPa). However, Group-1 (Unmodified ERA) exhibited the highest bacterial survival count (0.65 ± 0.11 CFU/mL) and the lowest μTBS (10.95 ± 0.55 MPa).

**Conclusion:**

Ery-doped ZrO_2_NPs and Ery-doped SepNPs modified adhesive presented satisfactory antimicrobial activity against Lactobacillus and μTBS as compared to the unmodified adhesive when bonded to primary dentin.

## INTRODUCTION

Partial caries removal (PCR) represents a tissue-conserving approach to managing dental carious lesions by removing irreversibly infected dentin while retaining remineralizable caries-affected dentin (CAD).^1^ While Streptococcus mutans initiates caries through acid production, Lactobacilli drive lesion progression in deeper cavities.^2^ The longevity of tooth-colored restorations depends on achieving durable adhesive bonding to dentin. However, CAD presents a compromised bonding substrate with reduced bond strengths due to altered collagen organization, decreased mineral content, and bacterial contamination.^3^ Conventional adhesives lack antimicrobial properties to neutralize residual bacteria, potentially increasing secondary caries risk.

Antimicrobial photodynamic therapy (aPDT) integrated with adhesive resins has emerged as a strategy for imparting antibacterial functionality to bonding agents.^4^ aPDT provides therapeutic advantages, including minimal thermal damage to tissues, negligible bacterial resistance, selective cytotoxicity toward microbes while preserving host tissue, and sustained antimicrobial effects.^5^ The mechanism involves light activation of a photosensitizer, generating reactive oxygen species (ROS) that damage bacterial cells through oxidative stress.^5^ Erythrosine (Ery), a xanthene-based photosensitizer absorbing at 530 nm, shows antimicrobial potential yet remains underutilized in adhesive dentistry.^6^ However, erythrosine’s hydrophilicity limits bacterial membrane penetration, prompting interest in nanocarrier systems for enhanced delivery.^7^

Nanotechnology integration into dental adhesives enhances multiple properties simultaneously. Zirconium oxide nanoparticles (ZrO_2_NPs) show exceptional biocompatibility and mechanical properties, improving micro-tensile bond strength (μTBS) to dentin through polymer matrix reinforcement.^8^ Sepiolite nanoparticles (SepNPs), fibrous clay minerals with high aspect ratios (20-30:1) and surface hydroxyl groups, provide reinforcement efficiency and antimicrobial properties.^9^ While research has explored photosensitizer-nanoparticle combinations in dental adhesives,^10^ the pairing of erythrosine with ZrO_2_ or sepiolite nanocarriers remains unexplored. Understanding these materials’ interactions within adhesive matrices and their impact on antimicrobial efficacy and bonding performance to CAD is crucial for developing multifunctional dental adhesives.

The present investigation evaluated whether incorporating erythrosine-loaded nanoparticles into etch-and-rinse adhesives (ERA) could simultaneously enhance antimicrobial effectiveness against Lactobacillus and improve bonding performance to primary CAD. The study compared conventional ERA with three experimental modifications: erythrosine alone (Ery-ERA), erythrosine-doped ZrO_2_NPs (Ery-ZrO_2_NPs-ERA), and erythrosine-doped SepNPs (Ery-SepNPs-ERA). The null hypotheses tested were: (1) no significant differences in antimicrobial efficacy among formulations; (2) no differences in μTBS to primary CAD.

## METHODOLGY

Sample size determination was performed using G*Power software (version 3.1.9.7, Heinrich Heine University, Düsseldorf, Germany). Effect size (Cohen’s f) = 0.40, alpha error probability (α) = 0.05 for a 95% confidence level, desired statistical power (1-β) = 0.80, and four experimental groups. 10 specimens per group were included, yielding a total sample size of 40 molars.^11^ This in vitro study was conducted in accordance with the ethical principles outlined in the Declaration of Helsinki and received approval from the Institutional Review Board and Ethical Committee of Princess Nourah, Binte Abdul Rahman University, IRB# 25-0063; dated November 19, 2025. The study duration was two months, from 20^th^ November 2025 to 20^th^ January 2026.

### Inclusion criteria and Preparation for study samples:

Forty primary molars with carious lesions that involved one-third of the dentin without pulp exposure (Score 5 according to the ICDAS system) were included. Each of the participants gave informed consent. Teeth were included if they presented: (1) carious lesions extending into the middle third of dentin; (2) Coronal structure without fractures or cracks; (3) Complete root formation with closed apices; and (4) no restorative treatment. For 48 hours at 4°C, all the included teeth were immersed in a disinfectant solution consisting of 0.5% Chloramine T (Sparchem, Mumbai, India). The specimens were prepared by eliminating any attached dental debris, plaque, and calculus, using an ultrasonic scaler (Esco Medco, Jiangsu, China). Using a dental explorer and a caries detection dye solution (CDS, Kuraray Co., Ltd., Osaka, Japan).^12^

### Lactobacillus culture and Growth kinetics:

The growth kinetics of Lactobacillus species (ATCC 393) were investigated over 52 hours using the pour plate enumeration method. Bacterial cultures were standardized to an initial optical density of 0.8–0.9 at 600 nm (OD_600_), corresponding to approximately 1 × 10^7^ to 1 × 10^8^ colony-forming units per milliliter (CFU/mL), as determined by spectrophotometric measurement. Standardized Lactobacillus inocula were aseptically transferred to sterile culture vessels containing 50 mL of de Man, Rogosa, and Sharpe (MRS) broth and incubated under aerobic conditions at 37 ± 0.5 °C. Following incubation, colonies were enumerated using a colony counter, and bacterial concentrations were calculated and expressed as CFU/mL. Only plates containing 30–300 colonies were considered for counting to ensure statistical reliability. Growth curves were constructed by plotting log_10_ CFU/mL against time to determine the lag, exponential, stationary, and decline phases of bacterial growth.^13^

CFU/ml = Number of Colonies Counted x Dilution Factor / Volume of Sample Plated in ml Loading and characterization of Ery doped ZrO_2_NPs and Ery doped SepNPs: Erythrosine (Ery, FD&C Red No. 3, C_20_H_6_I_4_Na_2_O_5_, molecular weight 879.86 g/mol), zirconium oxide nanoparticles (ZrO_2_NPs, particle size <50 nm, 99.9% purity), and sepiolite nanoparticles (SepNPs) were procured from Merck KGaA (Darmstadt, Germany). For surface characterization, ZrO_2_NPs and SepNPs were fixed on aluminum stubs of a scanning electron microscope (SEM) (SM-IT800, JEOL Ltd., Tokyo, Japan), followed by sputter coating with a gold-palladium for 120 secs. SEM was operated at an accelerating voltage of 10 kV. For elemental distribution, the NPs were assessed for Energy Dispersive X-ray Spectroscopy (EDX).^14^

### Preparation of Ery-ZrO_2_NPs:

Zirconium oxide nanoparticles (100 mg) were dispersed in 50 mL of distilled water in a clean glass beaker using magnetic stirring at 500 rpm for 10 minutes at room temperature. The suspension was then subjected to probe ultrasonication (Heilscher UP200S, Ultrasonics, Berlin, Germany) at 50% amplitude for five minutes in pulse mode (five seconds on, two seconds off) to ensure complete deagglomeration of nanoparticles. Erythrosine stock solution was then added dropwise to the stirring ZrO_2_NPs suspension over a period of 10 minutes while maintaining constant stirring. Ultrasonication was performed continuously for four hours at 30% amplitude in pulse mode to facilitate the adsorption of erythrosine molecules onto the nanoparticle surface through electrostatic interactions and hydrogen bonding.^15^

### Preparation of Ery-SepNPs:

Identical procedure as explained for the preparation of Ery-ZrO_2_NPs previously was followed for preparing erythrosine-loaded sepiolite nanoparticles, with SepNPs (100 mg) used as the carrier material.^16^

### Preparation of modified adhesive formulations:

A commercially available fifth-generation etch-and-rinse adhesive system (Adper Single Bond 2, 3M ESPE, St. Paul, MN, USA) served as the base adhesive for all experimental modifications. This adhesive contains Bis-GMA (bisphenol A-glycidyl methacrylate), HEMA (2-hydroxyethyl methacrylate), dimethacrylates, ethanol, water, and a proprietary photo initiator system. Four distinct adhesive formulations were prepared:

### Control group (Conventional ERA):

Unmodified Adper Single Bond 2 used directly from the manufacturer’s bottle without any additions.

### Experimental Group-1 (Ery-ERA):

Erythrosine powder was incorporated into the base adhesive at a concentration of 0.1 wt% (1 mg Ery per 1 g adhesive). This concentration was selected based on preliminary studies demonstrating optimal antimicrobial activity without compromising polymerization.^17^

### Experimental Group-2 (Ery-ZrO_2_NPs-ERA):

Prepared erythrosine-loaded zirconium oxide nanoparticles were incorporated at 1wt% concentration (10 mg Ery-ZrO_2_NPs per 1 g adhesive), providing dual benefits of mechanical reinforcement and antimicrobial activity.^18^

### Experimental Group-3 (Ery-SepNPs-ERA):

Prepared erythrosine-loaded sepiolite nanoparticles were incorporated at 1 wt% concentration (10 mg Ery-SepNPs per 1g adhesive) to evaluate the reinforcing and antimicrobial potential of clay-based nanocarriers.^16^

### Antimicrobial Photodynamic Therapy Activation Protocol:

To clearly distinguish aPDT activation from routine adhesive polymerization, a sequential two-step light exposure protocol was established based on the distinct light intensity requirements for photosensitizer activation versus resin curing.

### Step 1:

Antimicrobial Photodynamic Activation (Pre-polymerization Phase)

### Step 2:

Adhesive Polymerization (Curing Phase)

Immediately following the aPDT activation step, all specimens across all three groups received standard polymerization to achieve complete adhesive curing.^17^

### Artificial aging of the samples:

Each cycle constitutes immersion in both baths for 60 secs. The transfer time gap between the two baths was set to be five seconds. The total number of cycles was 5000. Each sample was then carefully inspected for any signs of defect or debonding at the resin-dentin interface. If any issues were discovered, those specimens were excluded from further analysis.

### μTBS testing and fracture pattern Assesment:

Using a slow-speed diamond saw (IsoMet™, Buehler, Lake Bluff, IL, USA), the bonded specimens were carefully sectioned into beams measuring 0.9 × 0.9 mm. μTBS was gauged using a universal testing machine (UTM) (Triax Digital 50, Controls, Milan, Italy). The ends of each beam were affixed using cyanoacrylate adhesive (Zapit, Dental Ventures of North America, Corona, CA, USA) to metal plates that were specifically designed for this purpose. Each beam in the experiment underwent a tensile load of 500 N, which was applied at a crosshead speed of 1 mm/min. The load was continuously increased until the beam fractured. The maximum load at failure was measured in MegaPascals (MPa). The fractured surfaces of all beam specimens were collected and examined using a stereomicroscope (VHX-5000, Keyence Corporation, Osaka, Japan) at 50× magnification to classify failure modes.

### Statistical analysis:

The normality of the data was assessed by conducting a Kolmogorov-Smirnov test. For normally distributed data with equal variances, one-way analysis of variance (ANOVA) was employed to detect overall differences among the four experimental groups. When significant differences were identified (p<0.05), pairwise post-hoc comparisons were conducted using Post Hoc Tukey.

## RESULTS

**Fig.1:**
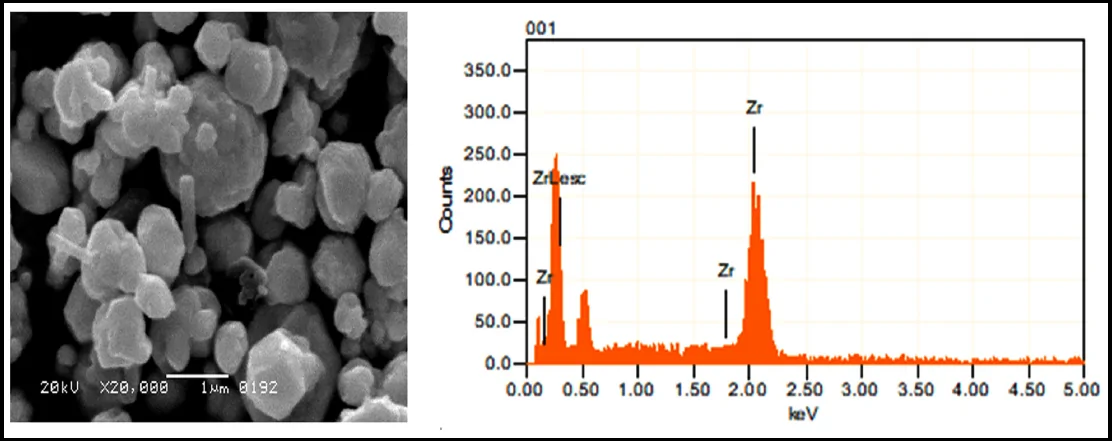
Surface characterization of ZrO NPs with EDX.

**Fig.2:**
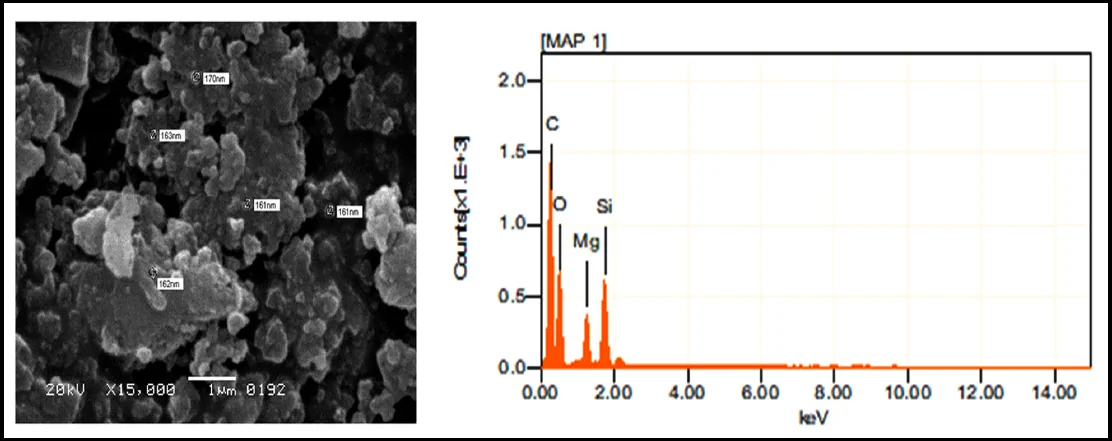
Surface characterization of SepNPs with EDX.

Surface Characterization and EDX Assessment of Sep and ZrO_2_ NPs: Fig.1: The SEM micrograph reveals that the ZrO_2_ nanoparticles exhibit a predominantly spherical to quasi-spherical morphology with some degree of irregular, faceted surfaces. EDX analysis shows Zirconium as a major component (78%), followed by O_2_ (22%). Fig.2: The SEM micrograph of sepiolite nanoparticles reveals a predominantly irregular, angular to sub-angular morphology with plate-like and blocky particle shapes. EDX assessment shows Carbon (5%), Oxygen (55%), Silicon (25%), and Magnesium (15%) as major elements in sepioliteNPs.

### Antibacterial efficiency against Lactobacillus:

The lactobacillus survival rate after modifying ERA is presented in Table-I. Outcomes indicated that Group-4 (Ery doped SepNPs ERA) (0.30 ± 0.04 CFU/mL) treated samples displayed the minimum survival of tested microorganisms. However, Group-1 (Unmodified ERA) exhibited the maximum count of bacterial survival (0.65±0.11 CFU/mL). Comparison among different groups unveiled that Group-3 (Ery doped ZrO2NP ERA) (0.33 ± 0.05 CFU/mL) and Group-4 revealed a comparable count of bacterial survival. (p>0.05) On the contrary, Group-2 (Ery-ERA) presented significantly lower survival than Group-1, yet higher than Groups-3 and 4 (p<0.05).

**Table-I:** Lactobacillus survival rate after using photodynamic therapy and nanoparticle-modified adhesive.

Experimental groups	Mean±SD CFU/mL	p-value*
Group-1: Unmodified ERA	0.65 ± 0.11^a^	<0.05
Group-2: Ery-ERA	0.45 ± 0.09 ^c^	
Group-3: Ery doped ZrO_2_NPs ERA	0.33 ± 0.05 ^b^	
Group-4: Ery doped SepNPsERA	0.30 ± 0.04 ^b^	

* ANOVA Erythrosine (Ery), Sepiolite Nanoparticles (SepNPs), Zirconium oxide Nanoparticles (ZrO2NPs).

Different superscript lower-case alphabets denote statistically significant differences within the same column (p<0.05), Tukey Post Hoc.

### μTBS testing and Fracture pattern analysis:

**Fig.3:**
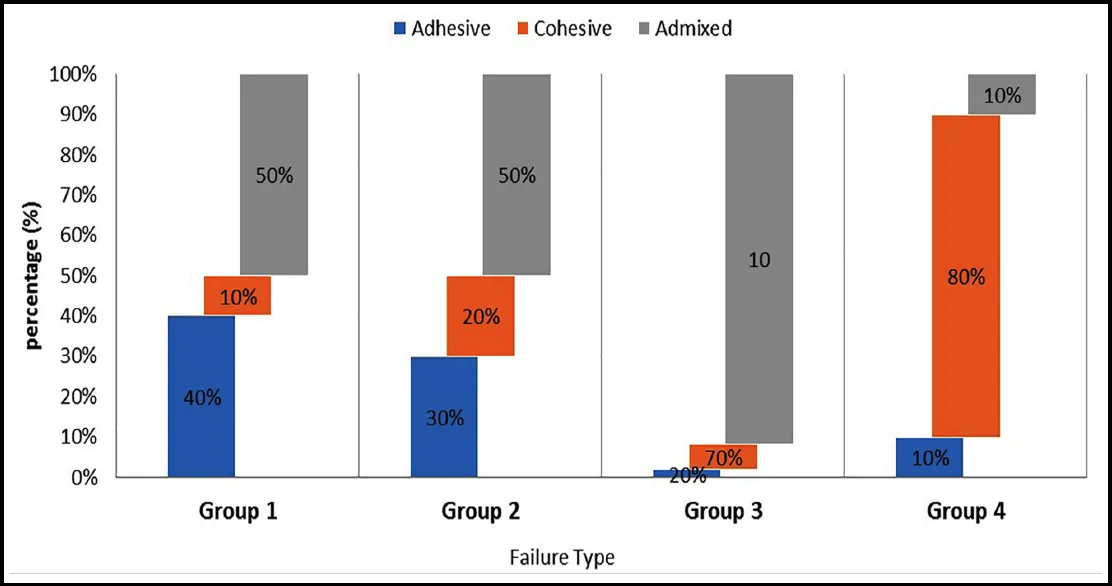
Percentage distribution of modes of failure.

μTBS values for ERA altered with Ery, SepNPs, and ZrO_2_NPs are displayed in Table-II. The study samples treated with Group-4 (Ery doped SepNPs ERA) unveiled the highest bond scores (15.02±0.61 MPa). In contrast, Group-1 (Unmodified ERA) samples exhibited the lowest μTBS (10.95±0.55 MPa). Compared to other groups, Group-3 (Ery doped ZrO_2_NPs ERA) (14.66±1.24 MPa) and Group-4 samples demonstrated no significant difference in bond strength results(p>0.05). However, Group-2 (Ery-ERA) (12.95±0.35 MPa showed significantly different results than all the other tested groups. (p<0.05). The percentage of failure modes in each experimental group is presented in Fig.3. Group-3 and Group-4 presented the cohesive failure type the most. However, it was identified that Groups 2 and 1 exhibited mixed types of fractures predominantly.

**Table-II:** μTBS after using PDT and nanoparticle-modified adhesive.

Experimental groups	(Mean ± SD) (MPa)	p-value*
Group-1: Unmodified ERA	10.95±0.55 ^a^	<0.05
Group-2: Ery-ERA	12.95±0.35 ^c^	
Group-3: Ery-ZrO_2_NPs -ERA	14.66±1.24 ^b^	
Group-4: Ery-SepNPs-ERA	15.02±0.61^b^	

* ANOVA Erythrosine (Ery), Sepiolite Nanoparticles (SepNPs), Zirconium oxide Nanoparticles (ZrO_2_NPs).

Different superscript lower-case alphabets denote statistically significant differences within the same column (p<0.05), Tukey Post Hoc.

## DISCUSSION

Both null hypotheses were partially rejected, as the experimental groups demonstrated superior antimicrobial efficacy and elevated μTBS values compared to the conventional adhesive. To the best of our knowledge, this study represents the first investigation into the integration of erythrosine-based photodynamic therapy, both in free and nanoparticle-loaded forms, into dental adhesive systems.

Regarding antimicrobial performance, the Ery-modified adhesive exhibited a Lactobacillus survival rate lower than the control but higher than both nanoparticle-enhanced groups. Upon photoactivation, erythrosine generates singlet oxygen and free radicals that interact with unsaturated organic compounds and fatty acids within bacterial membranes, causing oxidative damage and microbial inactivation.^19^ However, the relatively lower efficacy of Ery-ERA may be attributed to the suboptimal wavelength.^16^ The superior performance of Ery-doped ZrO_2_NPs-ERA can be attributed to the semiconductor properties of ZrO_2_NPs, which generate electron-hole pairs upon light exposure, producing superoxide anion radicals (O_2_-) that cause irreversible bacterial membrane disruption.^20^ Additionally, ZrO_2_NPs may facilitate intersystem crossing of erythrosine to the triplet state, enhancing energy transfer efficiency.^21^ For SepNPs, the high porosity, extensive surface area, active silanol groups, and fibrous morphology contribute to a “needle-stab effect” that physically disrupts microbial cytoplasmic membranes.^22^ Nevertheless, data on the synergistic effects of SepNPs with photosensitizers remain scarce and warrant further investigation.

Concerning bond strength, Ery-PS incorporation enhanced μTBS compared to the control, likely due to the electrostatic attraction between erythrosine’s superoxide ions and positively charged Ca^2+^ ions on the dentin surface, modifying the substrate to promote enhanced mechanical adhesion.^16^ The nanoparticle-loaded groups demonstrated even greater μTBS values, consistent with evidence that inorganic nanofillers function as shock-absorbing elastic layers at the composite–dentin interface, improving stress distribution.^9^ Their high surface area-to-volume ratio further enhances interfacial adhesion, with optimal SepNPs loading at 1 wt.% and uniform ZrO_2_NPs dispersion enhancing polymer network compatibility.

An important finding is the improved μTBS in nanoparticle-modified groups.^23^ The reactive surface chemistry of ZrO_2_NPs and SepNPs facilitates strong interfacial bonding through micromechanical interlocking and chemical coupling via surface hydroxyl groups, operating independently of polymerization extent.^24^,^25^ Furthermore, nanoparticles enhance fracture toughness through crack deflection and energy dissipation mechanisms, compensating for any reduction in cross-link density.^16^,^17^ Additionally, a moderately lower cross-link density may produce a more flexible polymer network that better accommodates polymerization contraction stresses, thereby reducing interfacial debonding.

Clinically, these findings are significant because residual bacteria beneath restorations remain a primary cause of secondary caries and restoration failure. The demonstrated antimicrobial efficacy against Lactobacillus, combined with improved bonding to the inherently challenging CAD substrate, suggests these modified adhesives could serve as therapeutic barriers at the resin–dentin interface. Importantly, the antimicrobial functionality is activated by the same LED curing light used routinely, requiring no additional equipment or procedural steps, which supports the translational feasibility of this approach.

### Limitations:

The in vitro design limits clinical extrapolation, as factors such as salivary dynamics, occlusal loading, and polymicrobial biofilm complexity were not simulated. Testing against a single bacterial species does not represent the multispecies biofilm environment. The use of one adhesive category and LED light rather than a wavelength-optimized green light source (532 nm) may have influenced the results.

### What the study adds to the current dental literature:

This study is among the first to introduce erythrosine-doped sepiolite nanoparticles as an etch-and-rinse adhesive modifier in caries-affected primary dentin, demonstrating simultaneous improvements in micro tensile bond strength and antimicrobial activity against Lactobacillus. The four-group comparative design isolates the contribution of the nanoparticle carrier from the photosensitiser alone, generating direct evidence that doped nanoparticle formulations confer superior adhesive and antimicrobial outcomes over erythrosine in its unmodified form.

### Study novelty:

The principal novelty of this study lies in the first application of erythrosine-doped sepiolite nanoparticles as a functional modifier of an etch-and-rinse adhesive for caries-affected primary dentin. By combining erythrosine’s established antimicrobial activity with sepiolite’s structurally distinct fibrous nanocarrier platform, the study introduces a dual-action adhesive strategy not previously explored in paediatric restorative dentistry.

## CONCLUSIONS

The incorporation of erythrosine-doped ZrO_2_NPs and erythrosine-doped SepNPs into a fifth-generation etch-and-rinse adhesive significantly enhanced both antimicrobial activity against Lactobacillus and micro-tensile bond strength to caries-affected dentin compared to the unmodified control.

### Recommendations:

Future investigations should employ multispecies biofilm models, long-term aging protocols with thermocycling, and optimized green light sources matching erythrosine’s absorption peak. Expanding these modifications to self-etch and universal adhesive systems would establish broader applicability.
